# Macrophage polarization in inflammatory bowel disease

**DOI:** 10.1186/s12964-023-01386-9

**Published:** 2023-12-21

**Authors:** Kun Zhang, Jing Guo, Wenlong Yan, Lingfen Xu

**Affiliations:** Department of Pediatrics, Shengjing Hospital of China Medical University, 36 Sanhao Street, Heping District, Shenyang, Liaoning 110004 China

**Keywords:** Macrophage, Polarization, Inflammatory bowel disease, Signaling pathway, Immunotherapy

## Abstract

The growing prevalence of inflammatory bowel disease (IBD) has encouraged research efforts, which have contributed to gradual improvements in our understanding of IBD diagnosis and therapeutic approaches. The pathogenesis of IBD has not been fully elucidated; however, the combined actions of environmental, genetic, immune factors, and microbial organisms are believed to cause IBD. In the innate immune system, macrophages play important roles in maintaining intestinal health and in the development of IBD. Macrophages can be polarized from M0 into several phenotypes, among which M1 and M2 play critical roles in IBD development and the repair of intestinal homeostasis and damage. Certain macrophage-related IBD studies already exist; however, the functions of each phenotype have not been fully elucidated. As technology develops, understanding the link between macrophages and IBD has increased, including the growing knowledge of the developmental origins of intestinal macrophages and their performance of comprehensive functions. This review describes macrophage polarization in IBD from the perspectives of macrophage development and polarization, macrophage changes in homeostasis and IBD, metabolic changes, and the mechanisms of macrophage polarization in IBD. The discussion of these topics provides new insights into immunotherapy strategies for IBD.

Video Abstract

**Supplementary Information:**

The online version contains supplementary material available at 10.1186/s12964-023-01386-9.

## Background

Inflammatory bowel disease (IBD), which is chiefly categorized as either ulcerative colitis (UC), Crohn’s disease (CD), or unclassified colitis, is a recurrent inflammatory disease with no recognized etiology [1]. The global prevalence of IBD has increased annually, reaching 84.3 per 100,000 people as of 2017. IBD has a significant impact on the quality of life of patients and increases the economic burden on governments [2].

Macrophages are one of the key effector cells involved in innate and adaptive immunity that remove dead cells and pathogens from the body during homeostasis, thereby inhibiting inflammation [3]. However, in patients with an immune disorder, excessive macrophage activation and infiltration can damage the intestine. The polarized M1 phenotype is thought to be directly associated with the onset of IBD, and anti-TNFα therapy can alleviate the disease by promoting macrophage M2 polarization [4–6]. Therefore, switching the phenotype of the macrophage is considered an effective therapeutic strategy [7–9].

Macrophages follow four primary activation pathways that generate macrophages with phagocytic, inflammatory, oxidative stress, or remodeling transcriptional states [10]. However, the role of macrophages in IBD has not yet been fully elucidated. The aims of this review are to determine the relationship between macrophage polarization and IBD and better understand the role of macrophage polarization in IBD. Particularly, this review explores macrophage origins, existing relevant markers, the relationship between macrophages and the intestinal epithelial barrier, metabolic alterations, and related molecules, as well as changes in macrophage polarization-related pathways and crosstalk between pathways. This review provides a theoretical basis for further research and treatment strategies for IBD.

### Macrophage development

**Fig. 1:**
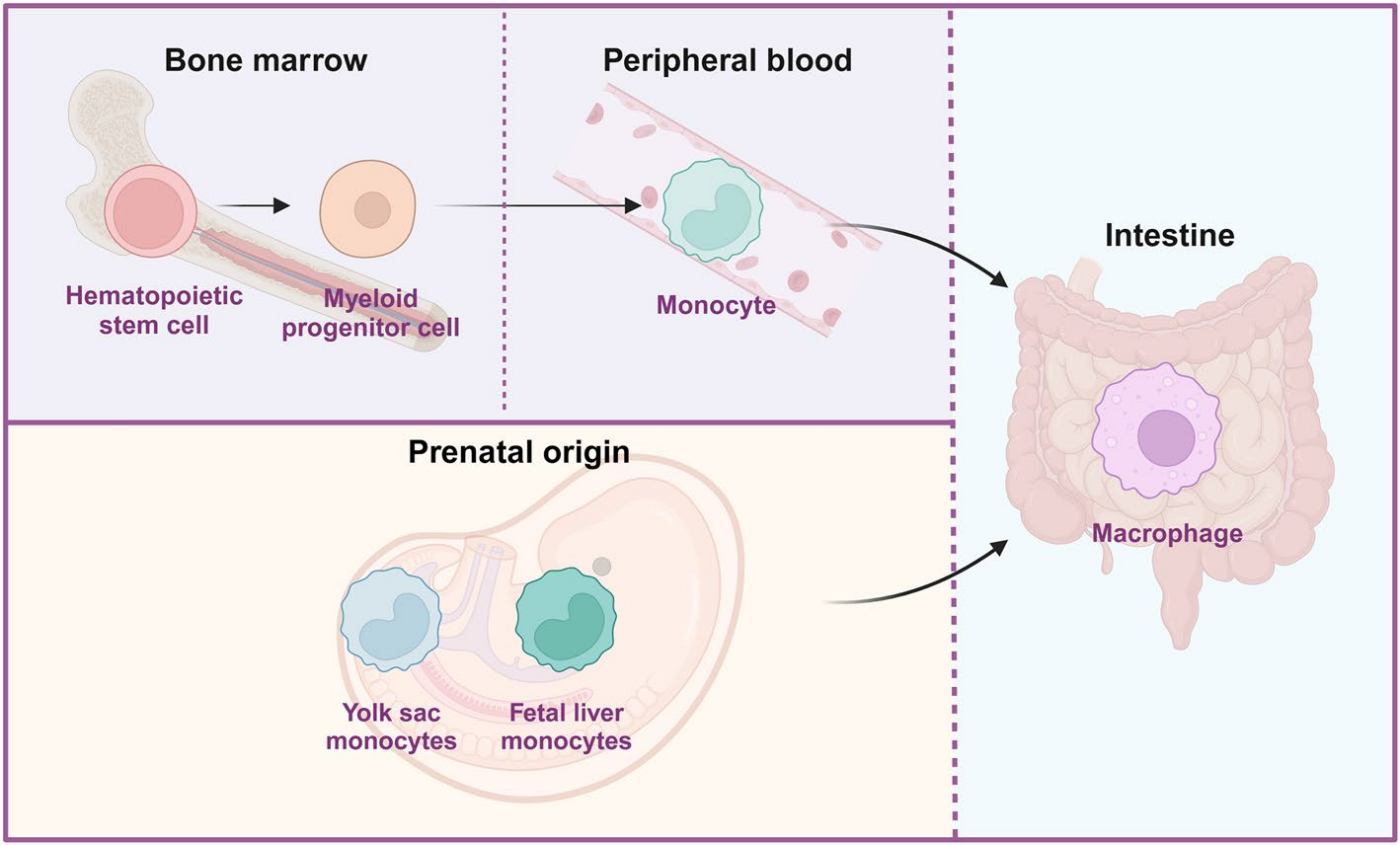
Origin of intestinal macrophages. (Created with BioRender.com)

Macrophages form part of the mononuclear phagocytic system and develop from the hematopoietic stem cells in the bone marrow, of which most develop into monocytes that are important for innate and adaptive immunity [11]. Bone marrow hematopoietic stem cells undergo continuous differentiation, evolving from progenitor cells to erythroid cells, megakaryocytes, bone marrow cells, and lymphocytes; bone marrow cells can then differentiate into monocytes and gradually mature in the blood [12, 13]. After a brief period in the bone marrow, monocytes enter the circulation and then the tissues, where they develop into macrophages [14]. Additionally, a small proportion of macrophages migrate to tissues throughout embryonic life and sustain elementary proliferation [15] (Fig. 1). Recent studies have revealed that the tissues surrounding macrophages undergo mitosis before birth, which reduces focal adhesion and macrophage infiltration, and may account for the entry of macrophages into the tissue during embryonic life [16]. Macrophages in different tissues have specific names, for example, bronchoalveolar macrophages in the lung tissue, microglial cells in the nerve tissue, osteoblasts in the bone tissue, and Kupffer cells in the liver tissue [15].

Macrophages, which are critical effector cells in the innate immune system, are abundant in tissues and essential for the regulation of intestinal homeostasis and inflammation, particularly in the gut [17]. Intestinal macrophages have two principal origins that exist and function together in intestinal tissues: resident macrophages present before birth and macrophages that are continuously replenished by circulating monocytes in adulthood [11, 18]. Originally, highly plastic circulating monocytes were thought to gradually replace postnatal resident macrophages over time. However, recent research has shown that a portion of embryo-derived macrophages, which exist in various anatomical ecological niches and play a corresponding supportive role, remain in the intestine, despite resident macrophages being continuously replenished by circulation during adulthood [17, 19]. Notably, macrophages play a crucial role in the creation, maintenance, and regeneration of new tissues in healthy organisms [17].

During inflammation, the number of resident cells decreases, circulating monocytes differentiate into tissue-resident cells to replenish tissue macrophages, and most circulation-derived macrophages retreat when inflammation subsides, although a small number of macrophages remain [10].

### Macrophage polarization phenotypes and markers

Macrophages play an important role as highly plastic innate immune cells in organisms [20]. Generally, macrophages polarize into M1 and M2 phenotypes, which are both involved in the development of IBD [21–23]. In addition, there exists an unpolarized state, called the M0 state. Previously, M0 macrophages in the gut have been incompletely characterized. Recently, Garrido-Trigo et al. [24] found that gut-resident macrophages can be classified into M0 and M2 phenotypes in healthy individuals. Moreover, the M2.2 clusters expressing M2 markers are closely related to the M0 phenotype and may represent a transitional state between M0 and M2.

**Fig. 2:**
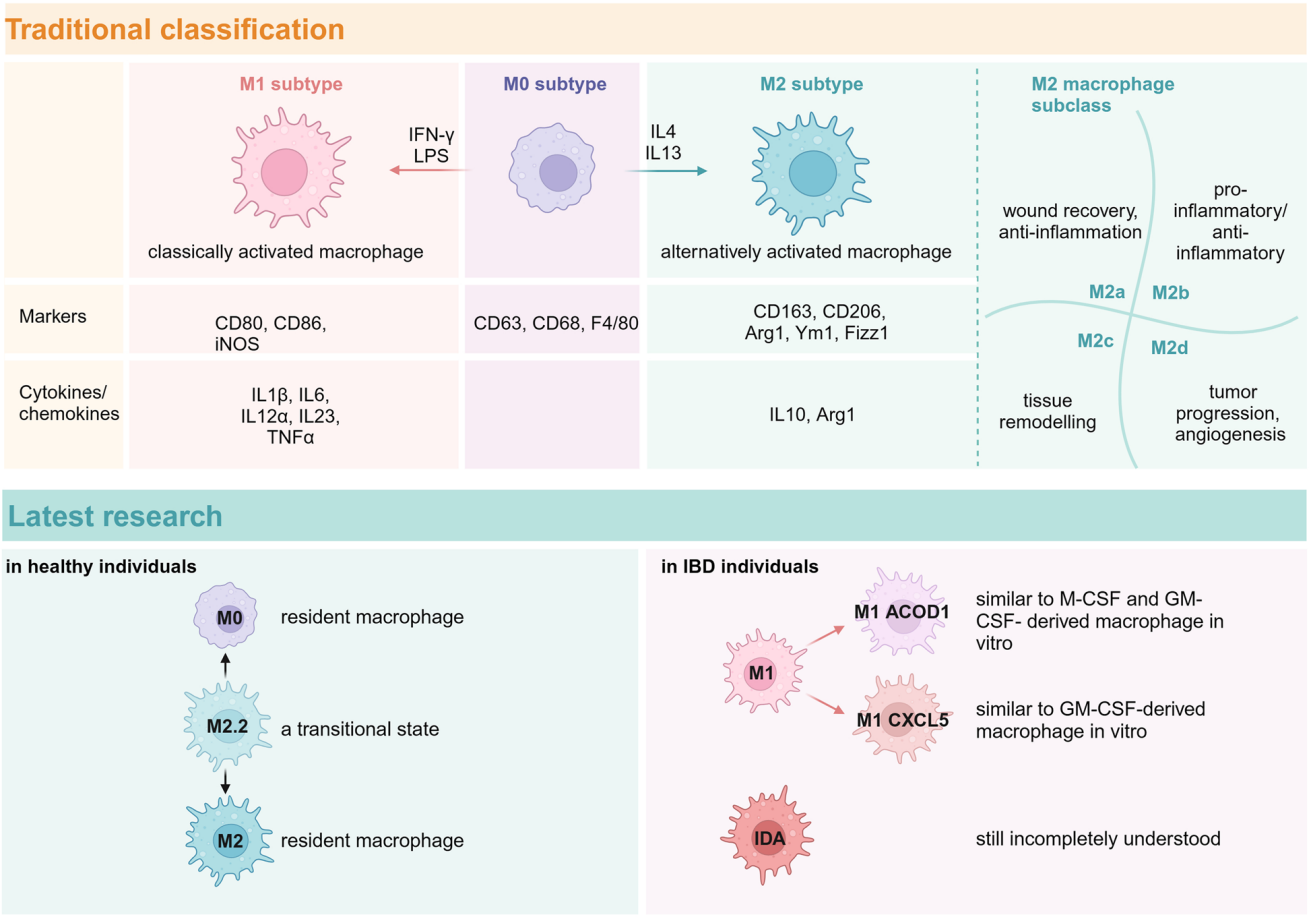
Macrophage polarization phenotypes, markers, and related factors. (Created with BioRender.com)

According to recent research, when macrophages are exposed to cytokines and other products in the microenvironment, they undergo macrophage polarization by differentiating into M1 or M2 macrophages with different phenotypes and functions [25]. Under certain conditions, M1 and M2 macrophages can transform into each other. For example, orally administered turmeric-derived nanovesicles in dextran sulfate sodium (DSS)-induced colitis mice accumulated in the inflamed mucosa and exerted good anti-inflammatory effects, and the RAW264.7 cell line showed a decrease in M1 macrophages but an increase in M2 macrophages, demonstrating that macrophages can remodel their phenotype and switch from M1 to M2 under certain conditions [8, 26] However, the reason for this transformation and its role in disease development have not been established [15]. Certain researchers believe that the balance between macrophage polarization and strong anti-inflammatory properties plays a more important role in the treatment of IBD than simply inducing the M2 polarization of macrophages [27]. These two distinct phenotypes produced by polarized macrophages produce different cytokines involved in different pathophysiological processes [28, 29]. M1 macrophages, also known as classically activated macrophages, can be induced by Th1 cytokines (e.g.,interferon-γ [IFN-γ]) and toll-like receptor (TLR) ligands (e.g., lipopolysaccharide [LPS]) and secrete pro-inflammatory cytokines such as interleukin(IL)-1β, IL-6, IL-12α, IL-23, and tumor necrosis factor(TNF)-α; thus, M1 is a pro-inflammatory phenotype [21, 25, 26, 30]. M2 macrophages, also known as alternatively activated macrophages, can be induced by IL-4 and IL-13, and secrete IL-10 and arginine(Arg)-1; thus, M2 is an anti-inflammatory phenotype that causes inflammation to subside and promotes the repair of tissue damage [21, 31, 32]. The M2 phenotype can be further divided into four subclasses: M2a, M2b, M2c, and M2d, which perform different functions. M2a macrophages are associated with wound recovery and anti-inflammation (after exposure to IL-4 or IL-13); M2b macrophages have been described as both pro- and anti-inflammatory (immune response to IL-1β or LPS); M2c macrophages exert tissue remodeling (immune response to IL-10); and M2d macrophages promote tumor progression and angiogenesis [33, 34]. In primary studies, general macrophages are commonly marked with CD63, CD68, and F4/80 [17, 35, 36]. For further differentiation, typical M1 macrophage-specific markers include CD80, CD86, and inducible nitric oxide synthase (iNOS), whereas M2 macrophage-specific markers include CD163, CD206, Arg1, chitinase 3-like 3 (Ym)1, and resistin-like-α (Fizz1) [21, 26, 29, 36–41] (Fig. 2).

Recently, Garrido-Trigo et al. [24] found that in patients with IBD, activated macrophages have at least three states known as M1 ACOD1, M1 CXCL5, and IDA macrophage clusters. Moreover, the different transcriptional profiles, from an inflammatory context, depend on different microenvironmental stimuli. For example, M1 CXCL5 cells are similar to GM-CSF-derived macrophages in vitro, and M1 ACOD1 cells are similar to M-CSF and GM-CSF-derived macrophages, and IDA macrophages are abundant throughout the inflamed colon; however, the origins and functions of IDA macrophages are still incompletely understood, and further studies are required [24] (Fig. 2). Hegarty et al. [3] summarized research related to intestinal single-cell sequencing and concluded that in humans and mice, macrophage heterogeneity depends on its distinct niche in the gut. They also found that differences anatomical location can have an effect on its phenotype, transcription, and function, further determining the different macrophage phenotypes [3]. Therefore, distinguishing between macrophages using M1 and M2 may no longer be appropriate.

### Macrophage polarization in intestinal homeostasis

#### Macrophages and intestinal barrier

**Fig. 3:**
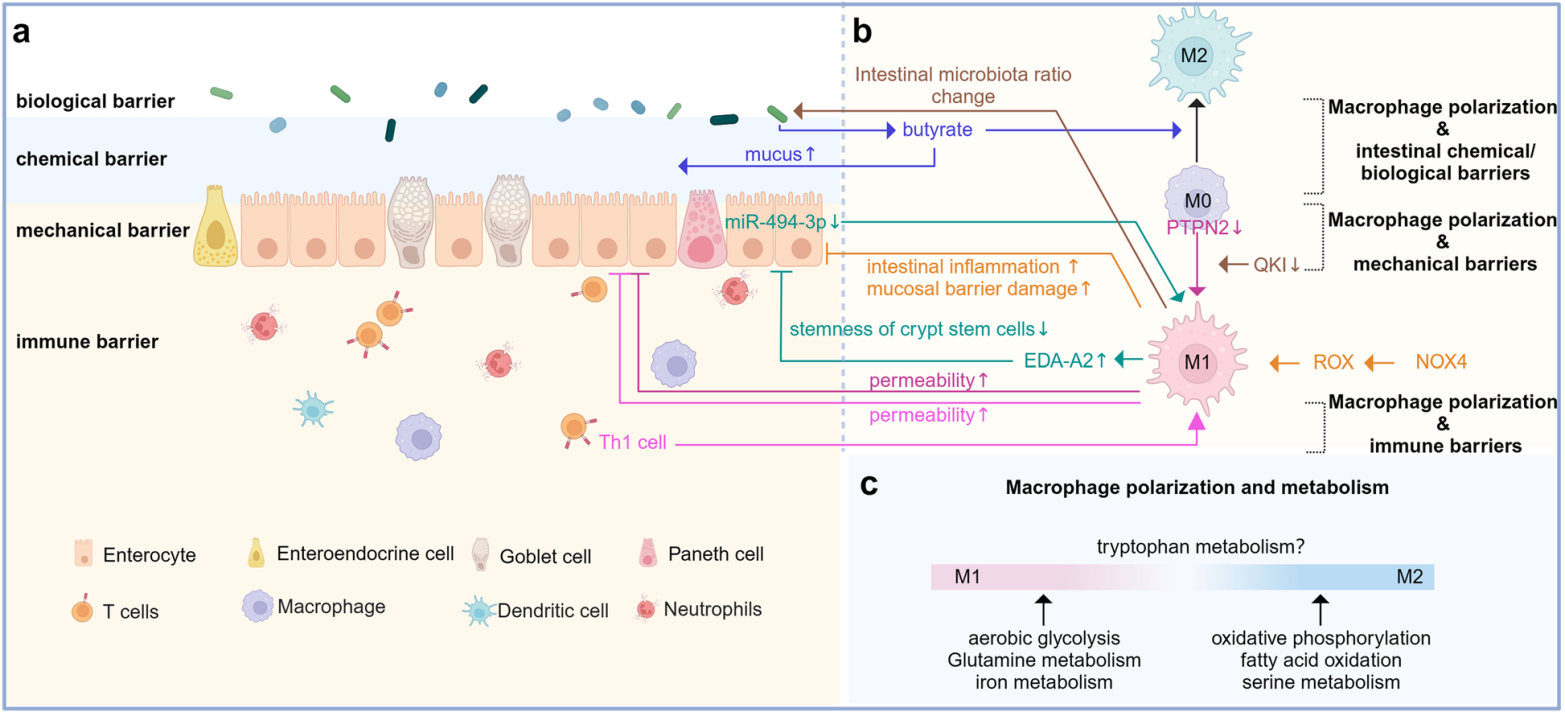
Macrophage polarization is correlated with intestinal barrier function and metabolism. **a** Diagram of the intestinal mucosal barrier; **b** Interaction between macrophage polarization and the intestinal mucosal barrier; and **c** Macrophage polarization and metabolism. (Created with BioRender.com)

The intestinal mucosa comprises epithelial cells, intraepithelial stroma, and immune cells [42]. An integral epithelial barrier prevents the activation of immune cells by undigested food proteins, microorganisms, and pathogens [42]. Intestinal homeostasis involves the intestinal mucosal barrier and metabolites generated by this barrier, which primarily include intestinal epithelial cells (IECs), intercellular junctions, intestinal epithelial secretions, intestinal flora, and immune cells, forming four major barriers: mechanical, chemical, biological and immune barriers [43] (Fig. 3a). Macrophages are abundant in the intestine and observed in the lamina propria, near the intestinal surface epithelium, and in the lumen of crypts [7]. Macrophage function is tightly regulated by microorganisms, and macrophages are more abundant in the colon, particularly the distal colon, than in the small intestine [44]. In a healthy body, intestinal macrophages are involved in various biological processes and are one of the primary contributors to the generation and preservation of intestinal homeostasis [45]. At this stage, macrophages predominantly belong to the M2 phenotype, exhibit a basal-tolerant state, and are capable of participating in the regression of inflammation and intestinal repair when stimulated [46]. Distal colonic macrophages can insert “balloon-like” protrusions between IECs, which can sample the fluid absorbed by the epithelial cells, thereby protecting them from fungal toxins and ensuring the integrity of the intestinal epithelial barrier [44]. When the intestine is stimulated by potentially dangerous luminal content, immune cells in the lamina propria switch to an activated state to protect the intestinal barrier in response to epithelial damage [47].

#### Interaction between macrophage polarization and the intestinal mucosal barrier

##### Macrophage polarization and mechanical barriers

The intestinal mechanical barrier primarily comprises of intestinal cells and intercellular junctions that block the entry of external bacteria, viruses, and toxins [48]. The expression of prostaglandin E receptor 4 in macrophages can promote the secretion of chemokine ligand (CCL) 1 and the subsequent differentiation and proliferation of crypt epithelial cells to regenerate and repair the damaged epithelium [49], suggesting a link between macrophages and IECs. Thus, macrophages regulate the integrity of the intestinal epithelium.

##### Macrophage polarization and intestinal chemical and biological barriers

The intestinal chemical barrier includes mucus and digestive juices secreted by IECs, as well as antibacterial substances secreted by beneficial intestinal bacteria, which can inhibit bacterial proliferation, dilute toxins, kill bacteria to some extent, and protect the intestine [50]. The intestinal biological barrier and gut microflora colonizing the intestinal surface constitute an interdependent and interacting ecosystem. Therefore, the intestinal chemical barrier is closely related to the intestinal flora [51].

##### Macrophage polarization and immune barriers

The intestinal immune barrier primarily comprises gut-associated lymphoid tissue, immune cells, and secreted immunoglobulin [52]. Macrophages, as cells of the immune system, are a crucial part of the mucosal barrier lining the digestive tract and prevent the colonization and adherence of intestinal pathogenic microorganisms [53].

### Macrophage polarization in IBD

Macrophages in healthy states can drive protective immunity against various attacks; however, excessive immune responses induce acute and chronic inflammatory diseases when the body is imbalanced [54]. For example, IL34 and colony-stimulating factor 1(CSF1) are important for macrophage maintenance, differentiation, and homeostasis, and share a common receptor, CSF1 receptor. During homeostasis, CSF1 preferentially differentiates monocytes into the M1 subtype, whereas IL34 shows preference for the M2 subtype. However, in IBD, both cytokines are highly expressed in tissues as pro-inflammatory agents, and the dual blockade of IL34 and CSF1 can significantly improve IBD symptoms [54–56].

#### The alterations in macrophages

Macrophages are important immune cells that remove bacteria and toxins during homeostasis; however, excessive activation of macrophages can trigger damage to the intestinal mucosal barrier, thus disrupting intestinal homeostasis and causing disease [42].

The dysregulation of intestinal macrophage homeostasis and the loss of antigen tolerance can result in the development of numerous inflammatory diseases, with IBD being a common example [57]. IBD is a category of recurrent inflammatory diseases that currently has no effective treatment owing to the diversity of its causes [58, 59]. Although the pathogenesis of IBD is unclear, available evidence from basic and clinical trials suggests that its onset, progression, and prognosis are related to immune imbalance [1]. Regarding innate immunity, intestinal macrophages in patients with IBD undergo important metabolic alterations, with excessive macrophage activation and infiltration leading to intestinal damage [7, 22], which suggest that macrophages are a major cell type involved in the pathogenesis of IBD [29]. In the inflamed intestine, macrophages are predominantly the M1 phenotype, which are capable of secreting pro-inflammatory factors, and together with the pro-inflammatory cytokines they produce, these macrophages contribute to the development of IBD [25].

In addition to a significant increase in M1 macrophages, a decrease in M2 macrophages is observed in patients with IBD [9]. Given the ability of macrophages to swap between phenotypes, several scientists have previously attempted to increase the proportion of M2 macrophages in IBD for therapeutic purposes [60]. However, recent studies have shown that M1 and M2 phenotypes are not mutually exclusive and both may be present in patients with IBD. The sequencing of macrophages from patients with IBD has revealed differential expressions of M2-related genes in patients with CD compared to those with UC, as well as significantly higher expressions of genes involved in CD fibrosis and granuloma formation (matrix metalloproteinase 12 and lysosome associated membrane protein 3) [22]. These results suggest that simply reprogramming intestinal M1 macrophages into M2 macrophages is not a rational therapeutic approach. This reprogramming not only fails to alleviate the recruitment and activation of T cells by M1 macrophages but may even exacerbate fibrosis and granuloma formation in CD [22]. Alternatively, treating IBD by increasing M2 macrophage polarization without promoting fibrosis-related severity might alleviate IBD symptoms to some extent. For example, exosomes from human bone marrow-derived mesenchymal stromal cells can promote macrophage M2 polarization, thereby alleviating mucosal inflammation without causing severe fibrosis in IBD [61]. Furthermore, an elevated M1/M2 macrophage ratio results in disease progression in IBD, tiliroside can degrade the hypoxia-inducible factor-1α proteasome and inhibit glycolysis, thereby inhibiting macrophage M1 polarization and improving the balance of macrophage polarization [27].

#### Interaction between macrophage polarization and the intestinal mucosal barrier

##### Macrophage polarization and mechanical barriers

In patients with IBD, protein tyrosine phosphatase non-receptor type 2 (PTPN2)-related variants exist in macrophages, and cellular experiments have shown that defects in PTPN2 can cause macrophages to polarize to the M1 phenotype, which can disrupt the IECs barrier when co-cultured with IECs, as evidenced by a decrease in transepithelial electrical resistance (TEER) and an increase in permeability [40]. IECs can also regulate the macrophage phenotype. In human IBD samples and in DSS-induced models of murine experimental colitis, miR-494-3p levels were reduced and negatively correlated with the severity of inflammation; thus, miR-494-3p is an essential protective agent in colitis. miR-494-3p deficiency in IECs is manifested by a significant increase in the expression of M1 macrophage polarization markers, the promotion of ectodysplasin A2 secretion by macrophages, inhibition of colonic crypt stem cell stemness, and impaired IECs repair [62]. NADPH oxidase 4 (NOX4) is one of the primary sources of reactive oxygen species (ROS) and increases nitric oxide synthases (NOS) expressions in IBD, which can promote the polarization of intestinal macrophages toward the M1 phenotype. In vitro experiments, the inhibition of NOX4 expression into co-cultured Caco2 cells and M1 macrophages resulted in decreased IECs barrier permeability and increased TEER values, which not only reduced epithelial cell death but also increased the expression of ligand proteins [63]. These results indicate that NOX4 can promote M1 polarization of intestinal macrophages via ROS to further aggravate intestinal inflammation and mucosal barrier damage in IBD [63]. Thus, macrophage polarization can interact with intestinal mechanical barrier integrity and plays an important role in the regulation of intestinal homeostasis (Fig. 3b).

##### Macrophage polarization and intestinal chemical and biological barriers

Several recent studies have identified important roles for the intestinal flora in IBD. First, intestinal microbiota-derived butyrate promotes macrophage M2 polarization. Second, upon application of butyrate in macrophages and goblet-like LS174T cell co-culture systems, the goblet cell markers mucin2 and SAM-pointed domain-containing ETS transcription factor were significantly increased and promoted mucus recovery after DSS-induced injury and reestablishment of the mucus barrier in mice [64]. Quaking (QKI) is an RNA-binding protein that can exert intestinal protective effects, and QKI-knockout mice showed elevated susceptibility to DSS and an increased proportion of M1 macrophages in the colon, with in vitro co-culture experiments suggesting that QKI deficiency could increase the level of NOS expression in epithelial cells. Moreover, according to fecal microbiota analysis, altered intestinal microbiota ratios and the fecal transplantation of QKI-knockout mice caused healthy mice to exhibit severe colonic damage [65], indicating an important role of intestinal flora in macrophage polarization. This result suggests that the interaction between macrophage polarization and the intestinal mucosal barrier is predicated on the presence of intestinal flora (Fig. 3b).

##### Macrophage polarization and immune barriers

Besides macrophages, research has also focused on immune cells, primarily T cells, in studies related to the pathogenesis of IBD [66, 67]. However, the mechanisms regarding the interaction between macrophage polarization and the immune barrier have not yet been fully elucidated. A recent study found that Th1 cells in a mouse model of IBD were able to increase mucosal barrier permeability by inducing macrophage M1 polarization [68] (Fig. 3b).

### Macrophage polarization and metabolism

Metabolism is involved in biological processes and plays an important role in the polarization phenotype of immune cells [69]. To elucidate if metabolism determines the macrophage phenotypes or whether macrophage polarization phenotypes determine metabolic status, numerous efforts have been made to determine the causal relationship between the two by demonstrating that alterations in specific cellular metabolism affect the macrophage phenotype and modulate the cytokine secretion profile at the level of bone marrow-derived macrophages [70]. The metabolic properties of M1 and M2 macrophages differ, and aerobic glycolysis is involved in macrophage M1 polarization, oxidative phosphorylation is involved in macrophage M2 polarization, and changes in metabolism have different effects on diverse macrophage phenotypes, which are reflected by a strong effect on the phenotype of M1 macrophages [70–72]. In IBD, Lee et al. [73] found that LMT503 was able to alleviate IBD by promoting macrophage reprogramming. They showed that LMT503 promoted macrophage M2 polarization, reduce catabolism, and promote anabolism, including mitochondrial metabolism, and this trial is now in a phase I study in humans [73]. However, the metabolism associated with macrophage polarization in IBD has still received relatively little attention. Therefore, this review explores the metabolic pathways associated with macrophage polarization in other diseases, investigates how these metabolic pathways affect macrophage polarization, and then speculates on the relevance of these findings to IBD pathogenesis.

M1 macrophages are often associated with enhanced glycolysis and an impaired tricarboxylic acid cycle, whereas M2 macrophages are correlated with enhanced oxidative phosphorylation and fatty acid oxidation; however, macrophage phenotypes cannot be simply distinguished as M1 or M2, and the metabolic pathways corresponding to these phenotypes are not specific. For example, in tumors, CD40 activates fatty acid oxidation and glutamine metabolism, promotes M1 polarization in macrophages, and exhibits antitumor properties, all without damaging the tricarboxylic acid cycle, suggesting that CD40 is capable of reprogramming macrophage metabolism through a glucose-non-dependent pathway [74]. The development of multi-omics has provided new ideas for studying metabolism and macrophage polarization. Combined proteomics and metabolomics analyses suggest that the glycolysis-related M1 phenotype and oxidative phosphorylation-related M2 phenotype may be generalizations, and that the link between metabolic alterations and macrophage polarization phenotypes is far more complex than originally thought [75].

In addition to arginine and glutamine metabolism, which are well understood in macrophages, we have summarized certain metabolic pathways associated with macrophage phenotypes, such as serine, lactate, iron, and tryptophan metabolism. For example, the inhibition of serine metabolism can enhance M1 polarization in macrophages while inhibiting M2 polarization [76]. Moreover, glutamine metabolism facilitates lactate synthesis, and the important role of lactate metabolism in immune cells was demonstrated in a study examining the pattern of caudal fin regeneration in zebrafish, where inhibited lactate metabolism impaired macrophage recruitment while inhibiting macrophage M1 polarization [77]. Furthermore, a rat model of lumbar stenosis revealed a correlation between iron metabolism and macrophage polarization, with an increase in iron pools observed in the disease model [78]. This exacerbated chronic inflammation in lumbar spinal stenosis and induced an inflammatory response due to M1 macrophage polarization both in vitro and in vivo, which was inhibited by the administration of melittin to suppress iron overload and regulate iron metabolism-mediated macrophage polarization [78]. Finally, tryptophan metabolism may play an intricate role in the shift between macrophage phenotypes and requires further investigation [75] (Fig. 3c).

### Mechanisms of macrophage polarization in IBD

Transcription factors, non-coding RNA, stem cells, extracellular vesicles, and signaling molecules play important roles in macrophage polarization in IBD.

#### Transcription factors and macrophage polarization

**Fig. 4:**
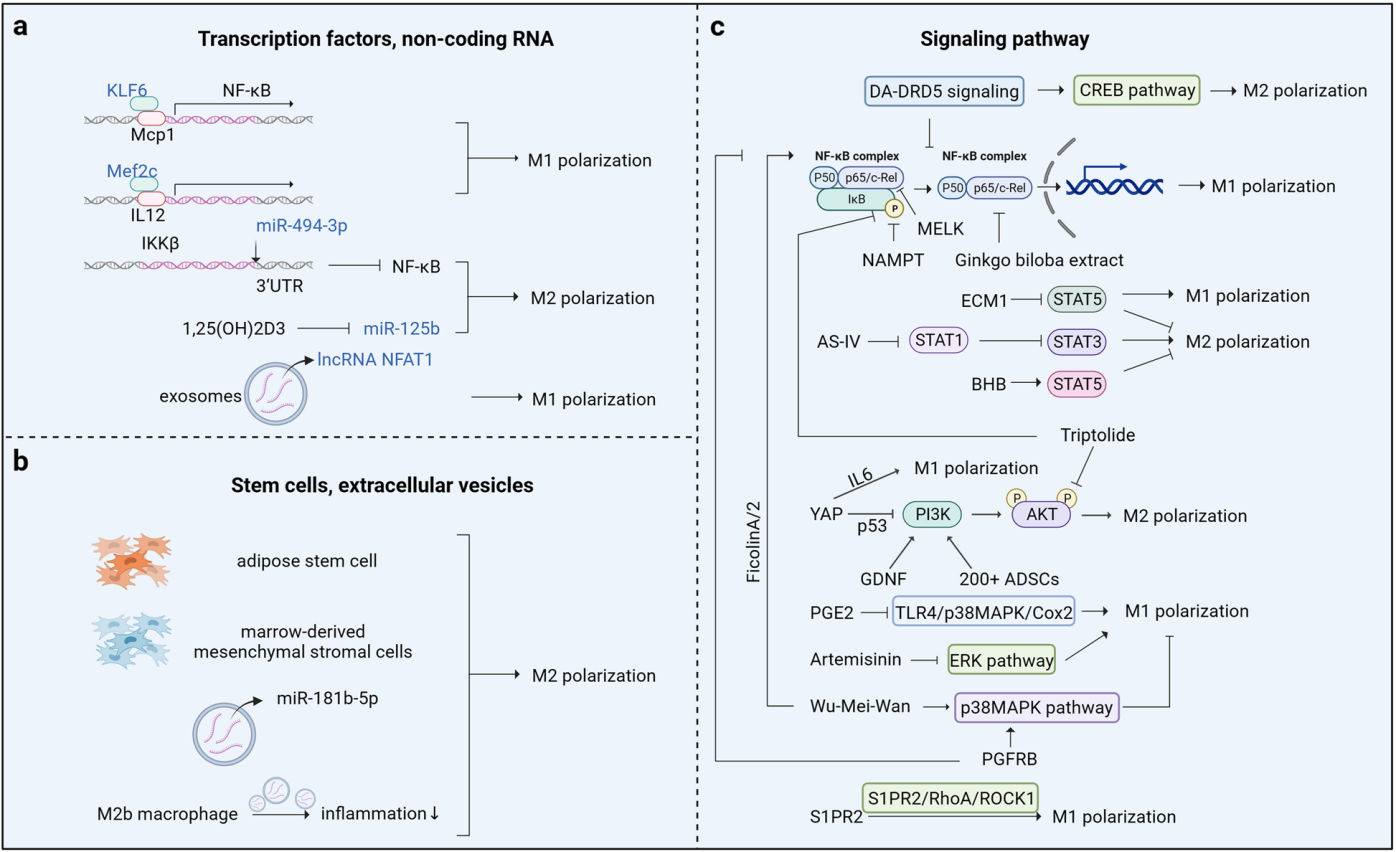
Mechanisms linked to macrophage polarization in IBD. **a** Non-coding RNA and macrophage polarization; **b** Stem cells and exosomes engage in macrophage polarization; and **c** Pathways related to macrophage polarization. (Created with BioRender.com)

Kruppel-like transcription factor 6 (KLF6) is the most abundantly expressed member of the KLF family [79]. KLF6 is highly expressed in humans and DSS-induced colitis, where it promotes the conversion of macrophages from a non-inflammatory phenotype to an M1 phenotype with increased expression of pro-inflammatory factors and decreased expression of anti-inflammatory factors [80]. Chip experiments have further demonstrated that KLF6 binds strongly to the macrophage chemoattractant protein 1 promoter, a pro-inflammatory target gene of nuclear factor-kappaB (NF-κB), after IFN-γ stimulation, suggesting that KLF6 promotes macrophage polarization toward M1 via the activation of NF-κB [80]. The myocyte-specific enhancer factor 2c (MEF2C) belongs to the MADS-box transcription factor family and has a highly conserved MADS and MEF2 domain at the N-terminus [25]. Deletion of MEF2C was observed in a DSS-induced mouse colitis model, resulting in downregulation of M1 macrophage marker expression, with dual luciferase reporter assays on macrophages demonstrating that MEF2C binds directly to the promoters of IL12a and IL12b to facilitate M1 polarization in macrophages [25] (Fig. 4a).

#### Non-coding RNA and macrophage polarization

MicroRNAs (miRNAs) are a class of small non-coding RNA consisting of 18–22 nucleotides that control gene expression and are involved in regulating cellular functions [81]. miRNAs are important for macrophage polarization [20]. The expression of miR-494-3p in differentiated IECs was decreased in the DSS-induced mouse model, and RNA-FISH demonstrated that miR-494-3p from IECs affected macrophage function [62]. For mimics of primary IECs transfected with miR-494-3p, decreased pro-inflammatory cytokines and macrophage-associated chemokines were observed [62]. Similarly, in the absence of miR-494-3p, pro-inflammatory cytokines and macrophage-associated chemokines were significantly elevated, suggesting that depletion of miR-494-3p in differentiated IECs promotes M1 polarization of macrophages in colitis [62]. The luciferase reporter gene assay results confirmed that miR-494-3p directly targets IKKβ 3’-UTR, and knockdown of IkappaB kinase-beta (IKKβ) revealed that a lack of miR-494-3p resulted in inactivation of NF-κB signaling, thus suggesting that miR-494-3p inhibits M1 polarization by targeting IKKβ/NF-κB signaling [62]. Furthermore, 1,25-dihydroxyvitamin D3 plays a protective role in colitis in DSS-induced mice and promotes LPS-induced conversion of M1 macrophages to the M2 phenotype via the downregulation of miR-125b [81] (Fig. 4a).

Long non-coding RNAs (lncRNAs) are transcripts > 200 nucleotides in length that play an important role in regulating cell proliferation, differentiation, and tumor formation [82]. Among the lncRNAs, nuclear paraspeckle assembly transcript 1 (NEAT1) was highly expressed in serum exosomes in a DSS mouse model, and the inhibition of NEAT expression in a cell line reduced the ability of TNF-α to promote M1 polarization, while promoting M2 polarization and inhibiting the inflammatory response [83]. Although many lncRNAs have been implicated in the development of IBD, the relationship between lncRNAs and macrophage polarization in this disease requires further investigation [84, 85] (Fig. 4a).

#### Macrophage polarization associated with stem cells and extracellular vesicles

Stem cells are a highly promising therapeutic tool in IBD. Extracellular vesicles play a role comparable to that of stem cells, and the proteins, mRNAs, and miRNAs encapsulated within these vesicles target specific parts of the colon or interact with specific cells through the vesicle structure to exert a therapeutic effect on IBD [86, 87]. Therefore, stem cell and extracellular vesicle therapies are emerging as promising therapeutic approaches for IBD.

Recently, adipose stem cells have been shown to play a role in the transformation of macrophage phenotypes. CD200 + adipose stem cells have been identified by single-cell sequencing, and their immunomodulatory function has been confirmed via co-culture with macrophages. In vivo administration reduces colonic inflammation in a DSS mouse model, and its derivative growth arrest specific 6 promotes macrophage M2 polarization via Mer/PI3K/Akt/GSK3β [88]. In addition, stimulation of canine adipose mesenchymal stem cells by cytokines produces secreted vesicles that exert stronger anti-inflammatory properties and enhance the modulation of T cells at the site of inflammation in colitis, thereby inducing macrophage activation toward the M2 phenotype and suppressing pro-inflammatory features [89]. Similarly, exosomes from human bone marrow-derived mesenchymal stromal cells alleviate colitis by promoting macrophage M2 polarization [61] (Fig. 4b).

The high expression of CCL1 in M2b macrophage exosomes reduces the expression of pro-inflammatory cytokines, which can promote Th2 polarization while increasing the percentage of Treg cells and alleviating inflammation through direct interaction with CCL8 [90]. Furthermore, miR-181b-5p in extracellular vesicles was highly expressed in an animal model of colitis, and a significant increase in M2 macrophage levels was observed with the addition of exogenous miRNA mimics in a DSS animal model [91]. This change was found to partially inhibit M1 polarization, reduce pro-inflammatory cytokine expression, and promote M2 polarization in an LPS-induced RAW264.7 cell model, resulting in reduced inflammation [91] (Fig. 4b).

#### Pathways of macrophage polarization in IBD

##### NF-κB signaling pathway

NF-κB comprises p50, p65, and inhibitory IkappaB (IκB) in a homodimeric or heterodimeric complex. Under normal conditions, these protein complexes are inactive in the cytoplasm. When the trigger factor activates the receptor, IκBα is degraded and p60/p65 is released into the nucleus to activate the target gene [92]. This pathway is divided into classical pathways that regulate inflammatory immune responses and homeostasis in the intestinal epithelium and alternative pathways that regulate intestinal inflammation and epithelial microfold cell function [93]. Several IBD studies have demonstrated the role of the NF-κB pathway in promoting macrophage polarization. Nicotinamide phosphoribosyltransferase is significantly upregulated in IBD and has recently been identified as a marker of IBD severity in children; its inhibitor, FK866, inhibits the development of inflammation, suggesting that FK866 can inhibit M1 polarization by inhibiting NF-κB and orienting macrophage polarization toward M2 [94, 95]. Corticosteroids have been widely used in the treatment of IBD, inactivating NF-κB and promoting monocyte polarization to M2 macrophages [3]. Recently, certain drugs have been shown to regulate macrophage polarization by inhibiting the NF-κB pathway. For example, *Ginkgo biloba* extract is thought to have anti-inflammatory effects, with a dose-dependent reduction in the expression of pro-inflammatory factors observed in an IBD model. In an LPS-induced cell model, the *G. biloba* extract bilobalide reduced p65 accumulation in the nuclei and re-freed it to the extracellular compartment, suggesting that bilobalide inhibited M1 polarization by improving NF-κB pathway activation [60].

##### JAK/STAT signaling pathway

In IBD, JAK/STAT pathway activation promotes inflammatory responses [96]. The classical JAK/STAT signaling pathway plays an important role in macrophage polarization, where the classical activation of macrophage polarization requires the signal transducer of activators of transcription (STAT) 1 [66] and the alternate activation of macrophage polarization requires STAT3/STAT5/STAT6 [31, 38, 97, 98]. Currently, several JAK blockers have been used in the clinic, and Ma et al. [99] summarized the findings of Shiratori et al. [100] and found that tofacitinib may ameliorate the disease by affecting the human macrophage phenotype. The traditional herb astragaloside IV (AS-IV) alleviates symptoms in DSS-induced colitis mice, and promotes the downregulation of M1-related genes and their markers, as well as the upregulation of M2-related genes and their markers [37]. Phosphorylation of STAT1 is downregulated, and further molecular structural modeling confirms that AS-IV binds to the STAT1 pocket [37]. STAT1 signaling inhibits STAT3 pathway activation, suggesting that AS-IV regulates STAT3 signaling by inhibiting STAT1 activation and macrophage polarization by downregulating STAT1 [37]. Extracellular matrix protein 1 (ECM1) is a susceptibility gene for IBD and is highly expressed in DSS colitis models. ECM1 deficiency in macrophages increases the susceptibility of mice to DSS, increases Arg-1 expression after granulocyte-macrophage colony-stimulating factor (GM-CSF) stimulation, activates the GM-CSF/STAT5 pathway, and impairs the M1 polarization process [31]. This suggests that ECM1 in IBD promotes M1 polarization by inhibiting the GM-CSF/STAT5 pathway while also inhibiting M2 polarization, and a series of inflammatory damage responses occur because of immune imbalance. In anti-TNFα studies, it is able to polarize macrophage M2 through the activation of IL10 signaling, of which JAK/STAT is a downstream molecule, and may further expand the mechanism of action of anti-TNFα therapies [100]. In addition, β-hydroxybutyrate promotes intestinal epithelial proliferation. According to a recent study, in an IL-4-induced co-culture system of M2 macrophages and the IECs line IEC-6, where the JAK2/STAT6 pathway was activated, the application of β-hydroxybutyrate resulted in significant proliferation of the IECs, which was completely inhibited by the STAT6 inhibitor AS1517499, suggesting that the proliferative effect of β-hydroxybutyrate on the intestinal epithelium is achieved via activation of the JAK2/STAT6 pathway in macrophages [57].

##### MAPK signaling pathway

The mitogen-activated protein kinases (MAPK) subfamily includes JNK, ERK, and p38MAPK, and studies related to experimental DSS-induced colitis have suggested that this pathway is associated with inflammation and apoptosis in intestinal tissues [101]. The inhibition of inflammation can be achieved by targeting prostaglandin E2 (PGE2); however, this is ineffective and may even be pathogenic in IBD [26]. PGE2 can inhibit macrophages polarization, and Th1 and Th17 infiltration, while promoting the proliferation of IECs in an IBD mouse model, suggesting a cell-specific effect of PGE2 [102]. Twist2-labeled stromal cells in the intestinal lamina propria express PGE2, and its rate-limiting enzyme cyclooxygenase-2 (COX2) is negatively correlated with the severity of IBD [102]. Moreover, the twist2 cell medium inhibits LPS-induced M1 polarization, and PGE2 inhibits M1 macrophage polarization and attenuates intestinal susceptibility to DSS or TNBS via the non-classical NF-κB pathway TLR4/p38MAPK/Cox2 [102]. Herbal preparations play an important role in the MAPK pathway, and artemisinin has positive effects on many diseases. In DSS-induced colitis, the extracellular signal-regulated kinase (ERK) pathway is activated, and M1 macrophages are abundant. After the administration of artemisinin, M1 macrophage-associated cytokines are downregulated, and the macrophage phenotype reverses to a predominantly M2 macrophage phenotype, whereas activation of the ERK pathway is inhibited [103]. Wu-Mei-Wan, a preparation used for the treatment of diarrhea, decreases the expression of pro-inflammatory cytokines and increases the expression of anti-inflammatory factors when administered to mice with DSS-induced colitis [39]. It also reduces inflammation by simultaneously inhibiting p38MAPK pathway activation and activating the STAT6 pathway, thereby inhibiting macrophage M1 polarization and promoting macrophage M2 polarization [39]. These studies suggest that activation of the MAPK pathway is involved in the macrophage polarization process, promoting M1 polarization and inhibiting M2 polarization in macrophages, resulting in IBD.

##### PI3K/AKT signaling pathway

The PI3K/AKT pathway regulates M2 polarization and inflammatory responses in macrophages, and many molecules affect IBD during this pathway. For example, yes-associated protein (YAP) was previously found to promote intestinal epithelial regeneration; however, studies on intestinal macrophages showed that deleting of the YAP gene in macrophages protects mice from colitis. YAP also inhibits M2 macrophage polarization by inhibiting the PI3K/AKT pathway. Further studies have revealed that this inhibition was achieved by YAP binding to the p53 promoter and promoting p53 expression. YAP also polarizes M1 macrophages and promotes IL-6 expression by binding to the IL-6 promoter [38]. In addition, glial cell line-derived neurotrophic factor (GDNF) is downregulated in DSS-induced colitis and during inflammation, and the use of GDNF reduces inflammation. Furthermore, GDNF converts macrophages to an M2 phenotype and reduces inflammation by activating the PI3K/AKT pathway in the RAW264.7 cell line after LPS induction [104]. In addition, CD200 + adipose-derived stem cells were recently identified as a new subpopulation of adipose-derived stem cells. This form of stem cell therapy effectively alleviates intestinal inflammation in experimental DSS-induced colitis by activating the Mer/PI3K/Akt/GSK3β pathway and promoting macrophage M2 polarization [105].

##### S1PR2/RhoA/ROCK1 signaling pathway

Sphingosine 1-phosphate (S1P) is an essential signaling molecule in sphingolipid metabolism, with S1P receptor (S1PR) binding involved in inflammatory responses. Previous studies have found that S1PR1 promotes macrophage M2 polarization [106] and S1PR3 promotes macrophage M1 polarization [107]. Furthermore, macrophage infiltration in an experimental DSS-induced colitis was accompanied by high S1PR2 expression, with the energy produced by the glycolytic process primarily used to supply M1 macrophages. The inhibition of key glycolytic enzymes following the use of S1PR2/RhoA/ROCK1 axis inhibitors suggests that S1PR2 promotes disease progression by promoting glycolysis to M1 polarization in macrophages [108].

##### Crosstalk between signaling pathways

In IBD, the pathways related to macrophage polarization are not separate. For example, FicolinA/2 stimulates M1 polarization through the TLR4/MyD88/MAPK/NF-κB pathway in macrophages, which exacerbates the inflammatory manifestations of IBD [35]. DA-DRD5 signaling inhibits macrophage M1 polarization by negatively regulating NF-κB signaling and promotes macrophage M2 polarization by activating the CREB pathway [109]. Platelet-derived growth factor receptor beta reduces the release of pro-inflammatory cytokines by activating AMPK and decreasing the expression of the NF-κB pathway and NOD-like receptor protein 3 (NLRP3) inflammatory vesicles [110]. *Bacillus cereus* exerts anti-inflammatory effects by regulating macrophage polarization and inhibiting the TLR4/NF-κB/NLRP3 pathway [111]. Moreover, triptolide inhibits DSS-induced colitis in vitro and in vivo, as shown by the downregulation of pro-inflammatory cytokines and upregulation of anti-inflammatory cytokines after drug application; in-depth mechanistic studies have revealed that triptolide significantly inhibits the phosphorylation of AKT and p65, as well as macrophage M1 polarization both in vitro and in vivo, by inhibiting PDEAB and thus regulating the PDE4B/AKT/NF-κB axis in macrophages [29]. Maternal embryonic leucine zipper kinase is upregulated in patients with IBD, and animal experiments have demonstrated that its pharmacological inhibition by treatment with OTSSP167 decreased the inflammatory response in DSS-induced mice [112]. According to the in-depth molecular mechanistic studies, this inhibitor can alleviate inflammation by inhibiting AKT/IKK/P65 and ERK/IKK/P65 signaling cascade responses both in vitro and in vivo [112].

## Conclusions

After years of research, our theories regarding the source of tissue macrophages have changed from abundant circulating monocytes to an embryonic source [17]. Simultaneously, the classification of macrophages has become less distinct with continued research, and with further evidence of their macrophage phenotypic and functional heterogeneity, distinguishing between inflammatory and anti-inflammatory processes is no longer appropriate [17]. Previous immunotherapeutic treatments for IBD have focused on reducing M1 macrophages and increasing M2 macrophages to reduce the damage caused by pro-inflammatory factors secreted by M1 macrophages in the intestine [113]. However, M2-related genes have a greater role than simply suppressing inflammation, and subsequent adverse manifestations, such as fibrosis and granulomas, suggest that simply switching between M1 and M2 phenotypes is not a beneficial therapeutic approach [22, 71]. Instead, sustaining a well-balanced ratio of polarized phenotypes or finding a solution to increase the number of anti-inflammatory phenotypic macrophages while mitigating the side effects associated with conventional therapies should be the focus of future research. The different roles played by IL34 and CSF1 in intestinal homeostasis and IBD also demonstrate that the functions of various factors vary with changes in body state. The current understanding of macrophage heterogeneity has gradually expanded, and classical phenotypic markers are no longer able to clearly distinguish different cellular states [20]. The number of metabolic pathways associated with polarized phenotypes has also increased from simple glycolysis and oxidative phosphorylation to a variety of complex metabolic pathways, further highlighting the complexity of the functions performed by macrophages in organisms. Macrophages are important immune cells in the intestine and can interact with the intestinal epithelial barrier through multiple pathways. Furthermore, the signaling pathways involved in macrophage polarization are intricate; many immunotherapies are not suitable for all patients, and the efficacy of treatments depends on patient specificity, thus requiring further in-depth studies [54]. Owing to recent advances in multi-omics studies and single-cell sequencing, which can be employed to detect differential genes, and with the development of stem cell and exosome technologies, individualized IBD treatment has become increasingly possible.

## Data Availability

Not applicable.

## Abbreviations

Arg: arginine
AS-IV: astragaloside IV
CCL: chemokine ligand
CD: Crohn’s disease
COX2: cyclooxygenase-2
CSF1: colony-stimulating factor 1
DSS: dextran sulfate sodium
ECM1: extracellular matrix protein 1
Fizz1: resistin-like-α
ERK: extracellular signal-regulated kinase
GDNF: glial cell line-derived neurotrophic factor
GM-CSF: granulocyte-macrophage colony-stimulating factor
IBD: inflammatory bowel disease
IECs: intestinal epithelial cells
IFN-γ: interferon-γ
IL: interleukin
IκB: IkappaB
IKKβ: IkappaB kinase-beta
iNOS: inducible nitric oxide synthase
KLF6: Kruppel-like transcription factor 6
lncRNAs: long non-coding RNAs
LPS: lipopolysaccharide
MAPK: mitogen-activated protein kinases
NEAT1: nuclear paraspeckle assembly transcript 1
ME2FC: myocyte-specific enhancer factor 2c
miRNAs: microRNAs
NF-κB: nuclear factor-kappaB
NLRP3: NOD-like receptor protein 3
NOS: nitric oxide synthases
NOX4: NADPH oxidase 4
PGE2: prostaglandin E2
PTPN2: protein tyrosine phosphatase non-receptor type 2
QKI: Quaking
ROS: reactive oxygen species
STAT: signal transducer of activators of transcription
S1P: Sphingosine 1-phosphate
S1PR: S1P receptor
TEER: transepithelial electrical resistance
TLR: toll-like receptor
TNF: tumor necrosis factor
UC: ulcerative colitis
YAP: yes-associated protein
Ym1: chitinase 3-like 3
